# Supplementary material for: Comparative genomics provides new insights into the diversity, physiology, and sexuality of the only industrially exploited tremellomycete: Phaffia rhodozyma
Source: BMC Genomics. 2016 Nov 9;17:901. doi: 10.1186/s12864-016-3244-7 (PMC5103461; doi:10.1186/s12864-016-3244-7)
Supplement: Additional file 6: — List of orphan genes with links to PFAM (related to Additional file 1: Table S1). (ZIP 1428 kb) [file 12864_2016_3244_MOESM6_ESM.zip › PFAM_UNIQUE/G01200_P.html]

#                                                                            --- full sequence --- -------------- this domain -------------   hmm coord   ali coord   env coord
  
# target name        accession   tlen query name           accession   qlen   E-value  score  bias   #  of  c-Evalue  i-Evalue  score  bias  from    to  from    to  from    to  acc description of target
  
#------------------- ---------- ----- -------------------- ---------- ----- --------- ------ ----- --- --- --------- --------- ------ ----- ----- ----- ----- ----- ----- ----- ---- ---------------------
  
G01200\_P             -            188 AT\_hook              PF02178.14    13     0.041   12.3  29.8   1   3   0.00053       0.4    9.3   5.5     1    12    32    43    32    44 0.88 - AT hook motif  
G01200\_P             -            188 AT\_hook              PF02178.14    13     0.041   12.3  29.8   2   3   0.00065      0.49    9.0   2.7     1    12    80    91    80    92 0.83 - AT hook motif  
G01200\_P             -            188 AT\_hook              PF02178.14    13     0.041   12.3  29.8   3   3    0.0093       6.9    5.4   5.9     3    10   180   187   180   187 0.91 - AT hook motif  
G01200\_P             -            188 BSP\_II               PF05432.6    292     0.039   11.9   1.7   1   1   0.00036     0.048   11.6   1.7    46    83   100   136    64   158 0.71 - Bone sialoprotein II (BSP-II)  
G01200\_P             -            188 Prothymosin          PF03247.9    106      0.29   10.0   4.5   1   1     0.004      0.53    9.2   4.5    41    83    95   135    82   154 0.63 - Prothymosin/parathymosin family
